# Synthesis of Functional Polyesters *N*-(2,3-epoxypropyl)-4,5,6,7-tetrahydroindole by Anionic Ring-Opening Polymerization

**DOI:** 10.3390/polym14204467

**Published:** 2022-10-21

**Authors:** Marina Markova, Artem Emel’yanov, Inna Tatarinova, Alexander Pozdnyakov

**Affiliations:** A.E. Favorsky Irkutsk Institute of Chemistry, Siberian Branch of the Russian Academy of Sciences, 1 Favorsky Str., 664033 Irkutsk, Russia

**Keywords:** *N*-(2,3-epoxypropyl)-4,5,6,7-tetrahydroindole, anionic homopolymerization, anionic copolymerization, ethylene glycol methylglycidyl ether, ethylene glycol vinylglycidyl ether

## Abstract

The functional polyethers of *N*-(2,3-epoxypropyl)-4,5,6,7-tetrahydroindole (in up to 61% yield, M_w_ = 8.7–11.7 kDa) and copolymers with ethylene glycol methylglycidyl ether (M_w_ = 5.6–14.2 kDa) and ethylene glycol vinylglycidyl ether (M_w_ = 6.4–12.3 kDa) have been synthesized via anionic ring-opening polymerization in the presence of KOH without solvent. The polymerization involves the opening of the epoxy ring to deliver the linear polyethers bearing free tetrahydroindole rings and oxyethylene or vinyloxy groups in the side chain. The polyethers are soluble in ethanol, benzene, chloroform, dioxane, DMF, and DMSO. The polyethers obtained exhibit the properties of high-resistance organic semiconductors: their electrical conductivity reaches 10^−14^ S/cm.

## 1. Introduction

Epoxy derivatives of heterocyclic compounds are valuable monomers for the design of high-tech polymeric materials. For instance, polymers of *N*-oxiranylmethylcarbazole are employed for recording and transmitting information. They are also used in devices that convert solar radiation energy into electrical one (solar photocells) and as photocapacitors [1,2,3,4,5,6,7]. Optical properties and film-forming ability of isolated carbazole molecules (photoluminescence maxima at 352 and 368 nm), compositions of low molecular weight epoxy resin Ruetapox 0162 with various amounts of 9-(2,3-epoxypropyl)carbazole and cured with isophoronediamine, can be exploited in LED fabrication [8]. Epoxy monomers can also be used to produce photopolymerizable compositions (PPC) in laser stereolithography with ultraviolet photoinitiating radiation [9].

Graft copolymers of *N*-epoxypropylpyrrole with polysiloxanes possess electrical conductivity in the range of 0.3–3.0 S/cm [10]. Copolymers of N-epoxypropylpyrrole with pyrrole, obtained by oxidative polycondensation, were claimed to be thin-film amperometric biosensors for urea [11] and galactose [12].

Polymers containing 4,5,6,7-tetrahydroindole and 1,2,4-triazole moieties in the side chain possess solubility, film-forming properties, and the ability to form complexes. The electrical conductivity of the obtained copolymers varies in a wide range from 10^−15^ S cm^−1^ to 10^−7^ S cm^−1^ (from dielectric to conductive material) depending on the composition and degree of doping with iodine [13].

Currently, only unsubstituted *N*-epoxypropylpyrrole is employed synthetic and applied research. The development of a general expedient method for the synthesis of pyrroles from ketoximes and acetylene in the presence of superbasic systems such as KOH/DMSO (Trofimov’s reaction) [14,15,16,17,18,19,20] opened the door to a plethora of pyrrole derivatives including 4,5,6,7-tetrahydroindole.

The improvement of the synthesis method of 4,5,6,7-tetrahydroindole [21,22,23], which makes it possible to obtain inexpensive THI on an industrial scale, accelerated the process of practical use of 4,5,6,7-tetrahydroindole derivatives. Thus, the prospects of using THI derivatives in the synthesis of drugs for the treatment of diseases such as Alzheimer’s disease and diabetes mellitus are shown [24]. THI derivatives are also promising in the treatment of oncological [25,26], viral [26], neurodegenerative diseases [27].

4,5,6,7-Tetrahydroindole is a “starting material” for the preparation of a new reactive class of epoxides, which are prospective building blocks for design of special polymeric semiconductors and photosensitive materials, intermediates of pharmacologically active compounds.

In the present work, we report for the first time the anionic homopolymerization of *N*-(2,3-epoxypropyl)-4,5,6,7-tetrahydroindole (EPTHI) and its copolymerization with ethylene glycol methylglycidyl ether and ethylene glycol vinylglycidyl ether.

The O–C–C–O bond sequence acts as a significant electron donor, providing polymers with polyoxide groups with hydrophilic properties such as solubility in many nonpolar organic solvents. In recent years, polyethylene oxides have become an object of scientific interest, such as drug delivery, tissue implantation and wound healing [28,29].

Over the last decade, polyethers, i.e., polymers containing the ether groups in the main chain (polyethylene oxides and polyalkylene oxides), have attracted much attention due to their possible applications as new safe electrolyte materials in energy storage devices such as lithium-ion batteries (solid polymer electrolytes). They are flexible, light, and non-flammable, they don’t leak [30,31,32,33].

The use of ethylene glycol methylglycidyl ether (EGMGE) and ethylene glycol vinylglycidyl ether (EGVGE) as comonomers enables to combine two important structural motifs, alkylpyrrole and oxyethylene (methyl ether or vinyl ether) in the desired polyether.

## 2. Materials and Methods

### 2.1. Materials

The IR absorption spectra of the synthesized polymers were recorded on a “Bruker Vertex 70” spectrometer (400–4000 cm^−1^) using films. ^1^H NMR spectra were recorded on a “Bruker DPX 400” (^1^H, 400.13 MHz) in CDCl_3_ using hexamethyldisiloxane as an internal standard. Number average molecular weight (M_n_) and molecular weight distribution (M_w_/M_n_) were determined by size exclusion chromatography using a Shimadzu LC-20 Prominence liquid chromatograph equipped with a differential refractive index detector. Separation was carried out on an Agilent PolyPore column (7.5 × 300 mm, granule size 5 µm, separation range 200–2 × 10^6^ g/mol) with a guard column in isocratic mode at 50 °C in *N*,*N*-dimethylformamide (eluent). The flow rate of the mobile phase was 1 mL/min, the sample volume was 20 µL. Polystyrene High EasiVials (Agilent, Santa Clara, CA, USA) standards with a narrow molecular weight distribution in the range from 162 to 6.6 × 10^6^ g/mol was used to construct the calibration curve. The data was processed using Lab Solutions software. Elemental analysis was performed on a FLASH 1112 series EA instrument from Thermo Finnigan (Parma, Italy). The electrical conductivity of the polymers was measured using a standard E6-13A teraohmmeter. The studied samples were prepared in the form of tablets by pressing under a pressure of 700 kg/cm^2^. Thermal analysis of polymers was carried out using a thermogravimetric analyzer TGA i1000 (Instrument Specialists Incorporated, Twin Lake, WI, USA) at a heating rate of 10 °C/min from 25 to 900 °C in air.

### 2.2. Methods

#### 2.2.1. Synthesis of *N*-(2,3-epoxypropyl)-4,5,6,7-tetrahydroindole

**Scheme 1:**
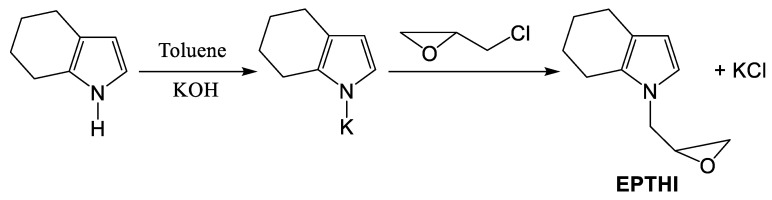
Synthesis of *N*-(2,3-epoxypropyl)-4,5,6,7-tetrahydroindole.

The reaction occurs through reaction of K-salt obtained in situ from KOH and 4,5,6,7-tetrahydroindole in a solution of toluene with epichlorohydrin, with azeotropic removal of water. The yields of *N*-(2,3-epoxypropyl)-4,5,6,7-tetrahydroindole were 68% [34] (Scheme 1):

#### 2.2.2. Synthesis of Ethylene Glycol Methylglycidyl Ether (EGMGE)

**Scheme 2:**
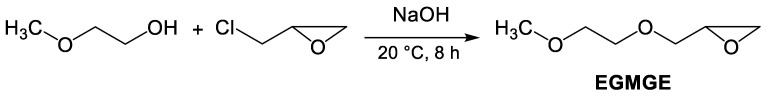
Synthesis of ethylene glycol methylglycidyl ether.

EGMGE was synthesized from ethylene glycol methyl ether and epichlorohydrin in 19% yield according to [35] (Scheme 2):

Ethylene glycol methyl ether (76.00 g, 1.00 mol) and NaOH (40.00 g, 1.00 mol) were placed into a flask equipped with a stirrer, dropping funnel and thermometer. After NaOH was partially dissolved, epichlorohydrin (138.00 g, 1.50 mol) was added slowly (dropwise) to the reaction mixture upon stirring, maintaining the temperature at 20–25 °C. At this temperature, the mixture was stirred for 8 h. Then the reaction mixture was diluted with ice water (80 mL) and was extracted with diethyl ether (100 mL). The ether was removed on a water bath and the product was fractionated in vacuo to give EGMGE (25.4 g, 19%), b.p. = 71 °C/10 mm Hg; n_D_^20^ = 1.4260. Found, %: C 53.88, H 8.82. C_6_H_12_O_3_. Calculated, %: C 54.54, H 9.09.

EGVGE was used in 99.9% purity (GLC control), the constants corresponded to the literature data: b.p. = 76 °C/6 mm Hg, n_D_^20^ = 1.4480, d_4_^20^ = 1.0333 [35]. The diethyl ether was purified by distillation.

#### 2.2.3. Anionic polymerization of *N*-(2,3-epoxypropyl)-4,5,6,7-tetrahydroindole (typical procedure)

EPTHI (0.56 g, 3.16 mmol) and powdered KOH (0.011 g, 2 wt.%) were heated (90 °C) for 14 h under argon atmosphere upon stirring. After cooling, benzene (0.3 mL) was added to the reaction mixture (viscous, tarry mass). The target polymer EPTHI **P7** was isolated by precipitation into hexane. The formed resin was washed with hexane and dried to constant weight to give hardened fragile dark-red resin with m.p. 127–130 °C (0.34 g, 61%), soluble in benzene, chloroform, ethanol, dioxane, DMF, and DMSO. Homopolymers **P1**–**P6**, **P8** were obtained similarly.

#### 2.2.4. Anionic copolymerization of EPTHI with EGMGE (typical procedure)

A mixture of EPTHI (0.0036 g, 0.0203 mmol), EGMGE (0.4964 g, 3.756 mmol), and powdered KOH (0.01 g, 2 wt.%) was heated (90 °C) under an argon atmosphere for 14 h upon stirring. The reaction mixture (colorless, transparent resin) was dissolved in benzene (0.3 mL) and precipitated into hexane to give copolymer **PM1** (0.42 g, 84%) as a viscous slightly yellow resin, soluble in benzene, chloroform, and DMF. Copolymers **PM2**–**PM5** and **PV1**–**PV5** (copolymers with EGVGE) were obtained similarly.

## 3. Results and Discussion

The presence of an epoxy group in the molecule *N*-(2,3-epoxypropyl)-4,5,6,7-tetrahydroindole opens up prospects for the use of EPTHI in polymerization and copolymerization with the epoxy ring-opening under anionic polymerization conditions.

### 3.1. Anionic Ring-Opening Polymerization of EPTHI

**Scheme 3:**
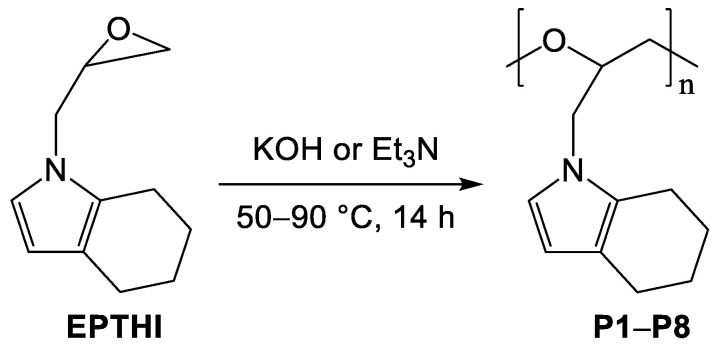
Anionic ring-opening polymerization of *N*-(2,3-epoxypropyl)-4,5,6,7-tetrahydroindole.

Anionic polymerization of EPTHI was carried out under argon (50–90 °C, 14 h) without a solvent in the presence of KOH (0.5–2 wt.%) or Et_3_N (2 wt.%) as catalyst. The reaction proceeds with the opening of the epoxy ring and the production of linear polyesters **P1–P8** with free tetrahydroindole rings in the side chain (Scheme 3):

The results of polymerization are presented in Table 1.

**Figure 1:**
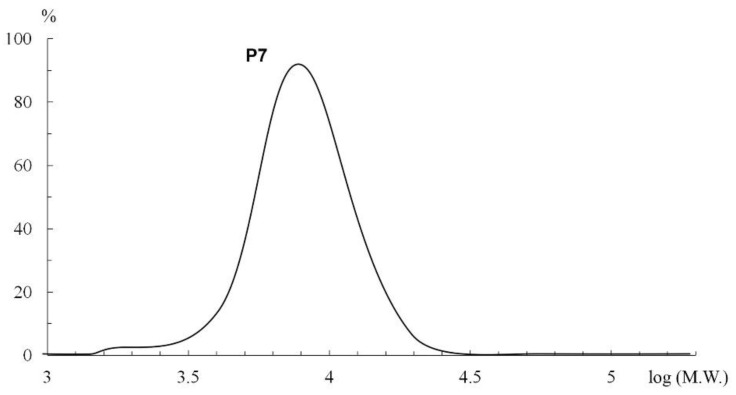
Typical curves of the molecular weight distribution (e.g., homopolymer **P7**).

The polyethers **P1**–**P8** obtained are brown resinous or powdered substances with a melting point of 127–130 °C, and molecular weight of 8.7–11.7 kDa (Figure 1). The polymers are soluble in ethanol, benzene, chloroform, dioxane, DMF, and DMSO.

**Figure 2:**
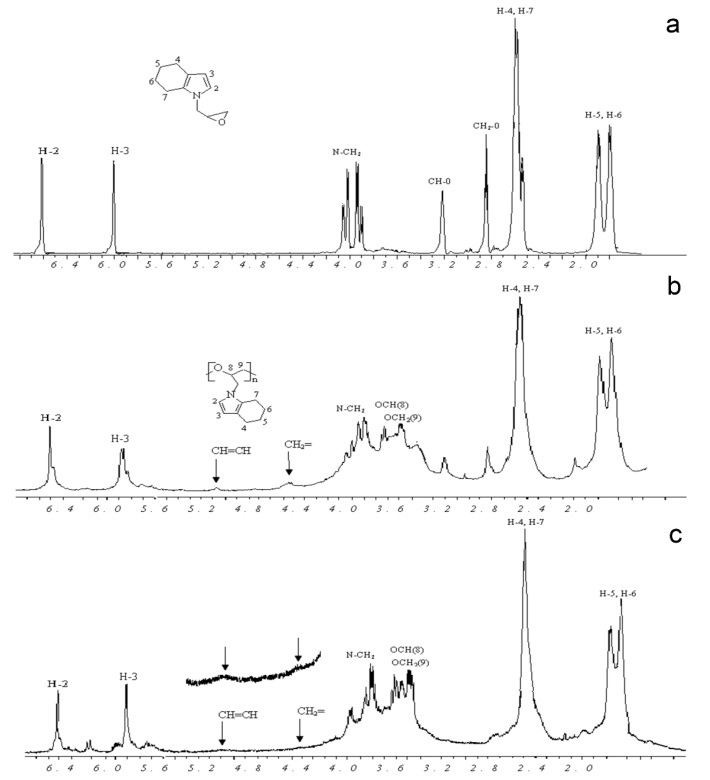
^1^H NMR spectra of EPTHI (**a**), polymer **P8** (**b**) and polymer **P7** (**c**).

The ^1^H NMR spectrum of the EPTHI is shown in Figure 2a. The ^1^H NMR spectra of the obtained polyethers (Figure 2b,c) show broadened signals characteristic of polymers. The signals of the pyrrole ring protons are observed at 6.55–6.45 ppm (H-2) and 6.00–5.50 ppm (H-3), while broad signals attributable to the CH_2_ groups of the cyclohexane ring are detected in the region of 2.50 ppm and 1.70 ppm. The signals at 3.82–3.40 ppm are assigned to CH-O, CH_2_-O groups of the main polymer chain and NCH_2_-pyrrole and the signals of CH=CH and CH_2_= groups appear at 5.11 ppm and 4.48 ppm.

**Scheme 4:**
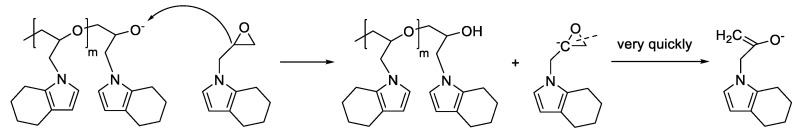
Formation of *cis*-propenyl end groups.

**Scheme 5:**
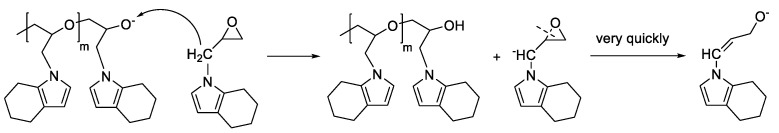
Formation of allyl-propenyl end groups.

The formation of the CH= and CH_2_= groups is caused by the chain transfer reaction to the monomer, which involves the abstraction of hydrogen from the CH group of the epoxy ring (Scheme 4) or from the alkyl substituent in the epoxy ring (Scheme 5) followed by very rapid ring cleavage to produce anions [36,37,38,39]:

The chain transfer leads to generation of new anionic active sites with a double bond. Interestingly, both *cis*-propenyl (Scheme 4) and allyl (Scheme 5) end groups can be formed.

In the IR spectra of polyethers **P1**–**P8**, the pyrrole ring bands remain at 708 cm^−1^ and 690 cm^−1^ (bending vibrations of the C–H group), 1298 cm^−1^ (stretching vibrations of the C–N group, characteristic vibrations for *N*-substituted pyrroles), 1370 cm^−1^ and 1490 cm^−1^ (stretching vibrations of the pyrrole core) [14]. The bands of the OH and ether groups appear at 3412–3365 cm^−1^, 1197–1254 cm^−1^ and 1136–1029 cm^−1^, respectively.

For polyether **P7**, synthesized at 90 °C using 2 wt.% KOH as a catalyst, weight average (M_w_) and number average (M_n_) molecular weights were determined (size exclusion chromatography) to be 8.9 and 7.1 kDa, correspondingly. The sample showed a unimodal molecular weight distribution (Figure 1). The polydispersity coefficient was 1.25.

As exemplified by polyether **P7**, EPTHI homopolymers exhibit the properties of high-resistance organic semiconductors: the value of their electrical conductivity is at the level of 10^−14^ S/cm (1.4·10^−14^ S/cm for **P7**). Doping with iodine vapor leads to resinification of the polymer that makes it impossible to measure the electrical conductivity.

### 3.2. Anionic Ring-Opening Copolymerization of EPTHI with EGMGE and EGVGE

**Scheme 6:**
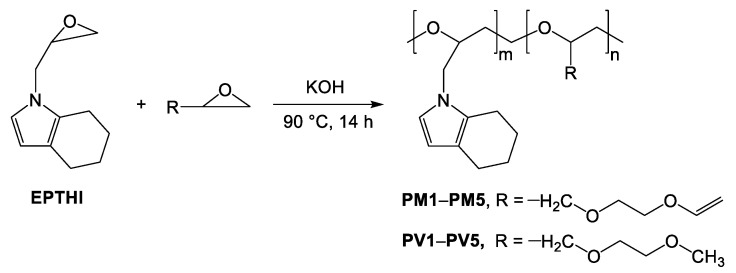
Anionic ring-opening copolymerization of EPTHI with EGMGE and EGVGE.

Anionic copolymerization of EPTHI with EGMGE and EGVGE was carried out under argon (90 °C, 14 h) without a solvent in the presence of KOH (2 wt.%) as a catalyst. The copolymerization occurs with the opening of the epoxy rings of both comonomers and the formation of soluble linear polyesters (Scheme 6).

The copolymers are resinous substances (soluble in benzene, chloroform, and DMF) with a molecular weight (M_n_) of 5.6–14.2 kDa and 6.4–12.3 kDa, respectively. The results of the copolymerization are given in Table 2.

In the ^1^H NMR spectra (CDCl_3_, δ, ppm) of copolymers **PM1–PM5**, together with the signals of the pyrrole ring protons (5.90 ppm and 6.50 ppm) and those of the CH_2_ groups of the cyclohexane ring (1.65–1.85 and 2.50 ppm) [13], the signals of EGMGE are detected at 3.35 ppm (CH_3_-O group), 3.80–3.50 ppm (CH-O and CH_2_-O groups of the main polymer chain and comonomer EGMGE). The signal of the *cis*-propenyl end group is observed at 5.11 ppm.

The composition of copolymer was calculated from the ratio of the integral intensities of protons of the pyrrole ring (5.87 ppm) and EGMGE CH_3_ group (3.35 ppm).

^1^H NMR spectra (CDCl_3_, δ, ppm) of copolymers **PV1–PV5** contain the following signals: 6.55 d (1H, H-2, J = 2.4 Hz), 6.4 q (1H, CH=, J = 6.84 Hz), 5.94 d (1H, H-3, J = 2.7 Hz), 4.22 dd (2H, CH_2_=, J = 2.20 and 14.32 Hz), 4.04 dd (2H, CH_2_=, J = 2.08 and 6.76 Hz), 3.9–3.5 m (4H, CH_2_-O, N-CH_2_ group), 2.57–2.40 m (4H, CH_2_-4, CH_2_-7), and 1.85–1.70 m (4H, CH_2_-5, CH_2_-6).

**Figure 3:**
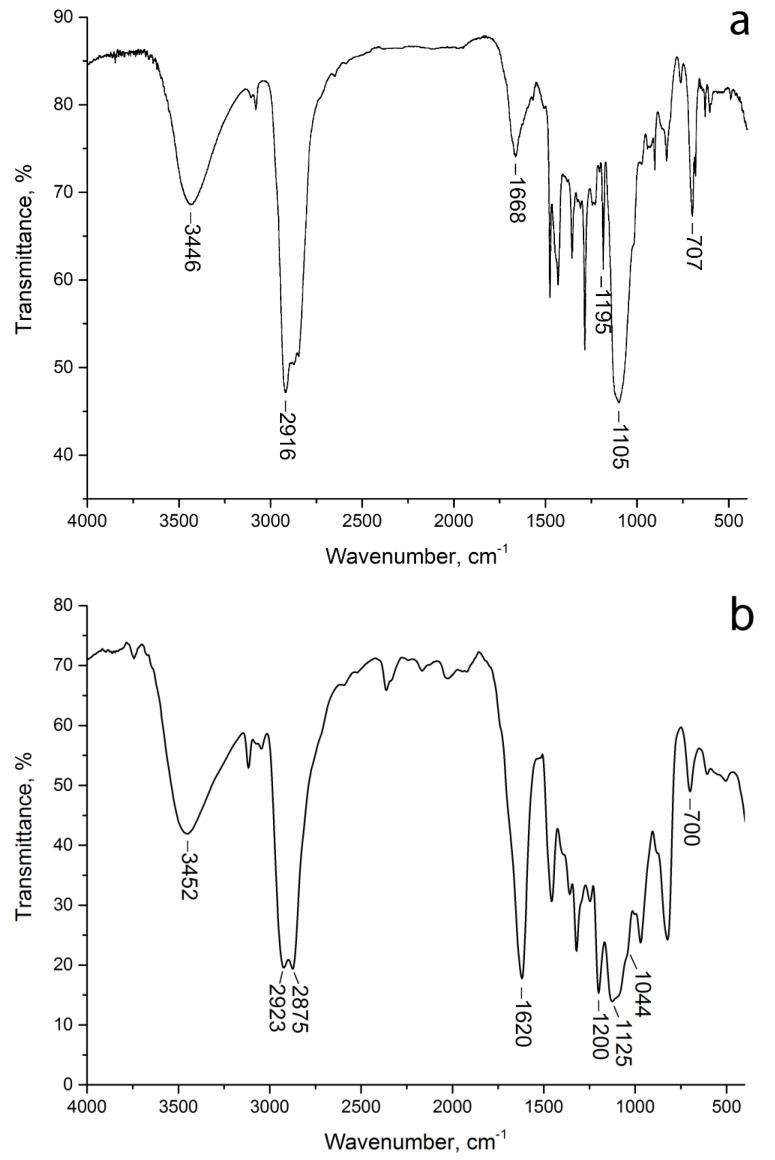
FT-IR spectra of copolymers **PM3** (**a**) and **PV3** (**b**).

The IR spectra of the copolymers show low intense bands of the terminal hydroxyl groups (3567–3364 cm^−1^ and 1200–1197 cm^−1^), probably due to the chain transfer to the monomer (Scheme 4 and Scheme 5), and the ether group (two bands in the region of 1125–1031 cm^−1^), bending vibrations of the C–H group of the pyrrole ring (700–755 cm^−1^). Intense absorption bands at 2931–2815 cm^−1^ correspond to stretching vibrations of hydrogen in the alkyl groups. In the spectra of copolymers, the absorption bands of the EGVGE vinyl group are observed at 3073 cm^−1^, 3046 cm^−1^, and 1620 cm^−1^ (Figure 3).

**Figure 4:**
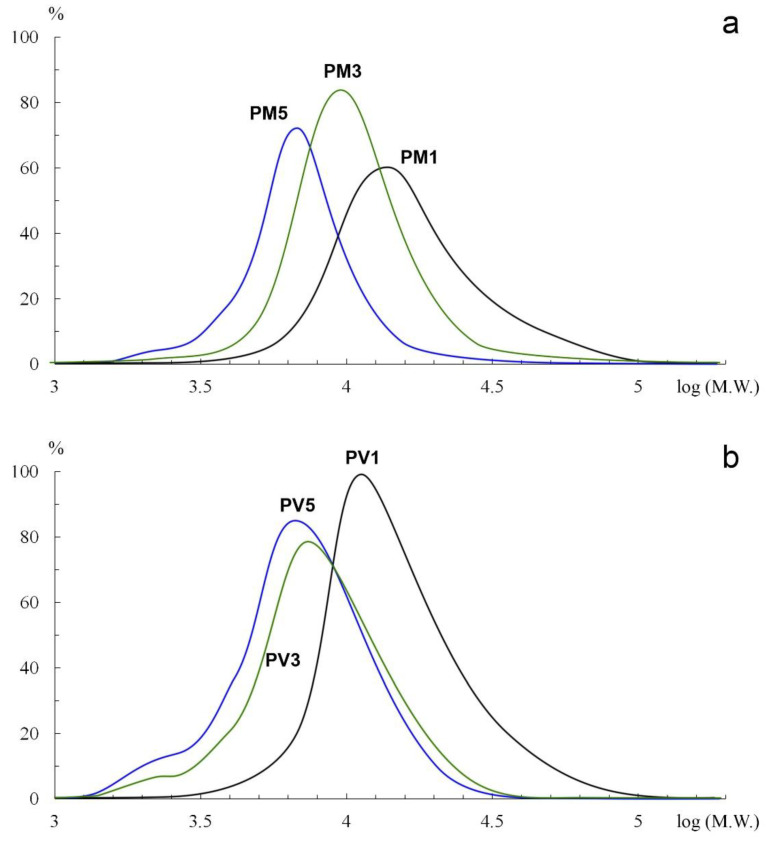
Chromatograms (curves) of the molecular weight distribution of copolymers **PM1**, **PM3**, **PM5** (**a**) and copolymers **PV1**, **PV3**, **PV5** (**b**).

The yield, structure, and molecular weight of the polymers depend on composition of the initial monomer mixture and reactivity of the comonomers. In copolymer **PM1** (Table 2), weight average molecular weight (M_w_) is 18.4 kDa, and number average (M_n_) is 14.2 kDa. When mass content of EPTHI increases to 0.9, the yield of polyether **PM5** decreases from 84% to 25%, M_w_ decreases to 8.9 kDa, and M_n_ reduces to 5.6 kDa, while the degree of polydispersity augments from 1.29 to 1.59 (Table 2, Figure 4).

For the system EPTHI and EGVGE, at higher content of the tetrahydroindole comonomer in the reaction mixture, the yield of copolymers **PV** increases from 58% (**PV1**) to 77% (**PV5**). The M_n_ of copolymers decreases from 12.3 to 6.3 kDa, and M_w_ reduces from 16.5 to 8.6 kDa, the degree of polydispersity remains almost unchanged (Table 2, Figure 4).

Chromatograms of the molecular weight distribution of copolymers **PM5** and **PV5** (Figure 4) containing more than 50% of EPTHI indicate the presence of up to 8% (for **PM5**) and 12–13% (for **PV5**) fractions with a molecular weight of 2.3 kDa. Sample **PV5** also has a fraction (up to 25%) with a molecular weight of 4.5 kDa. Polyethers **PM1**, **PV1** with mass content of glycidyl ethers of 0.9 have higher molecular weights (Table 2, Figure 4). These samples show a unimodal molecular weight distribution.

**Figure 5:**
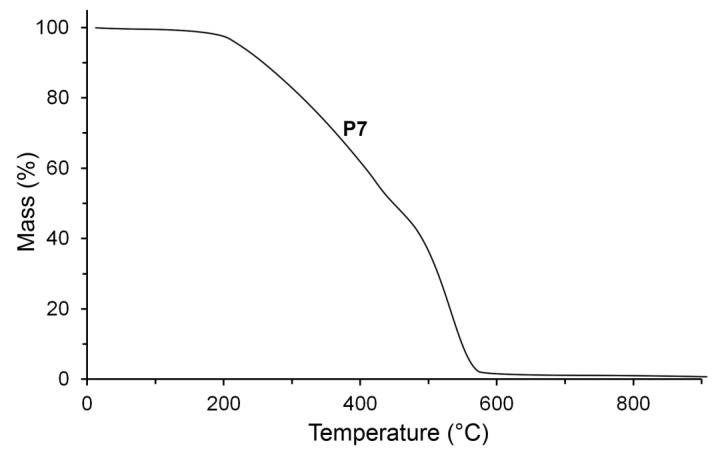
Thermal analysis of homopolymer poly-*N*-(2,3-epoxypropyl)-4,5,6,7-tetrahydroindole **P7**.

According to thermal analysis, polymer **P7** (Figure 5, Table 1) is stable up to 275 °C. Weight loss of sample **P7**, which occurs smoothly up to 489 °C, is 60%. This may be due to the abstraction of tetrahydroindole from homopolymer delivering polyfused heteroaromatic systems with loss of hydrogen and aromatization. This increases thermal stability of the polymer, which is reflected on the thermal destruction curve: the second phase of the macromolecule decomposition begins at 489 °C. Further up to 550 °C (in the range of 489–550 °C), the decomposition rate of sample **P7** becomes faster. In this temperature range, the weight loss is 35% (total weight loss reaches 95%). A further increase in temperature does not significantly affect the weight loss of polymer **P7** and at 947 °C the total weight loss is 99.71%.

**Figure 6:**
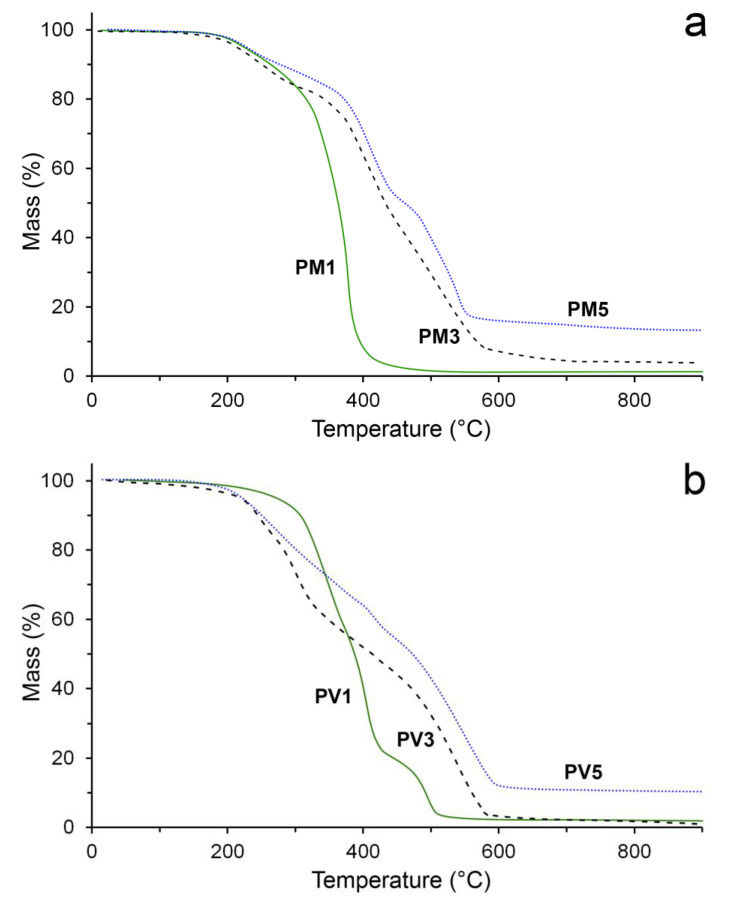
Thermal analysis of copolymers poly-*N*-(2,3-epoxypropyl)-4,5,6,7-tetrahydroindole with ethylene glycol methylglycidyl ether (**PM1**, **PM3**, **PM5**) (**a**), copolymers poly-*N*-(2,3-epoxypropyl)-4,5,6,7-tetrahydroindole with ethylene glycol vinylglycidyl ether (**PV1**, **PV3**, **PV5**) (**b**).

Copolymers containing EGMGE units (samples **PM1** and **PM5**) have a lower stability (270 and 260 °C) than polymer **P7**. Intense weight loss of copolymer **PM1,** containing only 5 mol.% of EPTHI, occurs up to 408 °C and is 90%. Probably, a significant part of the ethylene glycol methyl ether molecules is abstracted from the copolymer, which apparently contributes to the formation of a more thermodynamically stable polyconjugated polyvinylene chain (polyacetylene). A further increase in temperature to 600 °C leads to a smooth loss of sample weight (0.56% loss), apparently due to the elimination of residual fragments of ethylene glycol methyl ether and tetrahydroindole. The total weight loss at 600 °C is 99.56% (the weight of the sample is 0.44% of that taken for analysis). Weight loss of polymer **PM5** occurs in step-wise manner: at 378 °C it is 18%, then at 378–427 °C, weight loss is 27% (total weight loss reaches 45%). From 427 °C to 482 °C the sample decomposition rate decreases, the weight loss is 10% (the total loss reaches 55%). A further increase in temperature to 563 °C is accompanied by a 30% weight loss (the total weight loss reaches 85%). When the sample is heated to 717 °C, it does not significantly affect the weight reduction (weight loss is 2.44%, total weight loss is 87.44%). At the end of the study (at 717 °C), the weight of the sample is 12.56% of that taken for analysis (Figure 6a).

The presence of EGVGE units in the macromolecule (by eight times higher than those of EPTHI, sample **PV1**, EPTHI:EGVGE = 0.11:0.89) enhances the stability of the copolymer up to 310 °C. An increase in the number of EPTHI units (copolymers **PV3** and **PV5**) reduces the stability of the copolymer to 200–230 °C. At higher temperature (417 °C), the weight loss of sample **PV1** reaches 67% (total weight loss is 77%) (Figure 6b). For copolymers **PV3** and **PV5**, weight loss occurs smoothly and at 417 °C is only 42% and 29% (total weight loss is 52% and 39%, respectively). The complete decomposition of the copolymers occurs at about 500 °C (for sample **PV1**) and 600 °C (for samples **PV3** and **PV5**).

## 4. Conclusions

In conclusion, polyethers of *N*-(2,3-epoxypropyl)-4,5,6,7-tetrahydroindole have been synthesized for the first time in up to 61% yield and a molecular weight of 8.7–11.7 kDa by of anionic ring-opening polymerization with KOH or Et_3_N as the catalyst and without solvent. The polyethers are brown resinous or powdered substances, thermally stable up to 275 °C. EPTHI homopolymers exhibit the properties of high-resistance organic semiconductors: their electrical conductivity is at the level of 10^−14^ S/cm. The copolymers of EPTHI with EGMGE (yield up to 84%, M_n_ = 5.6–14.2 kDa, PDI = 1.29–1.59) and EGVGE (yield up to 77%, M_n_ = 6.4–12.3 kDa, PDI = 1.19–1.34) have been obtained. The polymerization involves the opening of the epoxy ring to produce linear polyethers with free tetrahydroindole fragments and oxyethylene or vinyloxy groups in the side chain. The polyfunctional reactive polymers and copolymers with tetrahydroindole and vinyloxy groups can be employed as macromonomers for further polymerization and copolymerization both at the pyrrole ring (cationic polymerization) and at the vinyloxy group (for copolymers with EGVGE). They provide additional opportunities for directed tuning of their properties through modification at the reactive pyrrole ring.

## Figures and Tables

**Table 1:** Synthesis conditions of *N*-(2,3-epoxypropyl)-4,5,6,7-tetrahydroindole.

Polymer	Catalyst, wt.%	T, °C	Yield, %
**P1**	KOH, 2	50	2
**P2**	KOH, 0.5	70	3
**P3**	KOH, 1	70	5
**P4**	KOH, 2	70	14
**P5**	KOH, 0.5	90	4
**P6**	KOH, 1	90	12
**P7**	KOH, 2	90	61
**P8**	Et_3_N, 2	70	30

**Table 2:** Anionic copolymerization of EPTHI (M_1_) with EGMGE and EGVGE.

Copolymer	Ratio of Monomers, mol.%		Yield, %	Content N, %	Composition of Copolymers, mol.%		M_n_, kDa	M_w_, kDa	M_w_/M_n_
	M_1_	M_2_			M_1_	M_2_			
EGMGE (M_2_) *									
**PM1**	10	90	84	0.50	5	95	14.2	18.4	1.29
**PM2**	25	75	65	2.79	29	71	12.1	16.1	1.33
**PM3**	50	50	50	4.28	47	53	10.0	13.7	1.37
**PM4**	75	25	34	4.32	53	47	7.9	11.3	1.43
**PM5**	90	10	25	4.88	56	44	5.6	8.9	1.59
EGVGE (M_2_) **									
**PV1**	10	90	58	1.08	11	89	12.3	16.5	1.34
**PV2**	25	75	63	1.87	20	80	10.3	12.2	1.19
**PV3**	50	50	65	3.25	36	64	7.8	10.2	1.32
**PV4**	75	25	67	4.06	46	54	6.4	8.5	1.33
**PV5**	90	10	77	4.46	51	49	6.4	8.6	1.34

* 2 wt.% KOH, 90 °C, 14 h; ** 2 wt.% KOH, 90 °C, 49 h.

## Data Availability

Not applicable.
